# Supplementary material for: Recent Achievements in Medicinal and Supramolecular Chemistry of Betulinic Acid and Its Derivatives
Source: Molecules. 2019 Sep 30;24(19):3546. doi: 10.3390/molecules24193546 (PMC6803882; doi:10.3390/molecules24193546)
Supplement: Supplementary file 1 [file molecules-24-03546-s001.pdf]

Supplementary Material

Recent achievements in medicinal and supramolecular chemistry of betulinic acid and its derivatives

Uladzimir Bildziukevich [1,2], Zülal Özdemir [1,2] and Zdeněk Wimmer [1,2,*]

[1] Institute of Experimental Botany of the Czech Academy of Sciences, Isotope Laboratory, Vídeňská 1083, 14220 Prague 4, Czech Republic; wimmer@biomed.cas.cz

[2] University of Chemistry and Technology in Prague, Department of Chemistry of Natural Compounds, Technická 5, 16628 Prague 6, Czech Republic; wimmerz@vscht.cz

Table S1. Physico-chemical and ADME parameters of the target compounds **15a**–**15c**, **20a**–**20c**, **22a**–**22c** and **24a**–**24c**

Compd. or recom. range	MW	Physico-chemical and ADME parameters [a]									
		log *P*	log *D* (pH 7.4)	log *S* (pH 7.4)	H_{acc} / H_{don} / n.m.b.	bioav. [%]	log *PS* * $f_{u,\ brain}$	log *PS*	log PB	log BB	PPB [%]
**15a**	498.78	7.77	3.34	–4.07	4/4/4	30-70	–5.8	–3.6	+0.2	+0.2	99.10
**15b**	524.82	6.09	5.47	–5.00	4/2/2	30-70	–3.9	–1.7	+0.35	+0.21	98.63
**15c**	641.02	6.09	0.17	–0.02	6/6/14	30-70	–5.2	–3.1	+0.66	+0.66	96.85
**20a**	598.86	5.20	2.70	–6.38	7/4/9	< 30	–4.2	–2.1	+0.06	+0.07	99.02
**20b**	624.89	9.13	3.65	–6.74	7/2/7	< 30	–6.6	–4.5	+0.0	+0.0	98.78
**20c**	741.10	5.49	1.48	–4.12	9/6/19	< 30	–5.2	–3.0	+0.61	+0.61	97.17
**22a**	646.90	7.56	4.78	–5.01	7/2/9	< 30	–5.8	–4.0	–0.89	–0.0	99.80
**22b**	646.90	7.56	5.26	–5.04	7/2/9	< 30	–5.8	–3.9	–0.89	–0.0	99.80
**22c**	646.90	7.56	5.07	–5.48	7/2/9	< 30	–5.8	–3.9	–0.88	–0.0	99.80
**24a**	546.83	7.00	7.00	–6.76	4/2/4	30-70	–4.1	–1.8	–0.30	–0.0	99.75
**24b**	546.83	7.00	7.00	–6.61	4/2/4	30-70	–4.1	–1.8	–0.30	–0.0	99.75

**24c**	546.83	7.00	6.99	–6.58	4/2/4	30-70	–4.1	–1.8	–0.30	–0.0	99.75
recom. range	180/500	–0.4/+5.6	–	–6.5/+0.5	10/5/–	–	–	–	–1.5/+1.5	–3.0/+1.2	–

[a] log *P* – partition coefficient; log *D* – distribution coefficient; log *S* – predicted aqueous solubility; H_{acc} / H_{don} / n.m.b. = number of H-bond acceptors / number of H-bond donors / number of movable bonds; bioav. = bioavailability – the degree of availability of a chemical by the target tissue; log $PS * f_{u, brain}$ – the brain/plasma equilibration rate, the parameter that is a mathematical modeling parameter based on time required for reaching brain equilibrium; log *PS* – logarithm of the permeability-surface area coefficient; log PB – the extent of brain penetration parameter; log BB – a hybrid parameter determined by permeability, plasma and brain tissue binding, and active transport mechanism; PPB – plasma protein binding.
